# Managing sleep and wakefulness in a 24-hour world

**DOI:** 10.1111/1467-9566.12046

**Published:** 2013-08-20

**Authors:** Catherine M Coveney

**Affiliations:** Department of Sociology, University of Warwick

**Keywords:** sociology of sleep, shift work, students, 24-hour living

## Abstract

This article contributes to literature on the sociology of sleep by exploring the sleeping practices and subjective sleep experiences of two social groups: shift workers and students. It draws on data, collected in the UK from 25 semi-structured interviews, to discuss the complex ways in which working patterns and social activities impact upon experiences and expectations of sleep in our wired awake world. The data show that, typically, sleep is valued and considered to be important for health, general wellbeing, appearance and physical and cognitive functioning. However, sleep time is often cut back on in favour of work demands and social activities. While shift workers described their efforts to fit in an adequate amount of sleep per 24-hour period, for students, the adoption of a flexible sleep routine was thought to be favourable for maintaining a work–social life balance. Collectively, respondents reported using a wide range of strategies, techniques, technologies and practices to encourage, overcome or delay sleep(iness) and boost, promote or enhance wakefulness/alertness at socially desirable times. The analysis demonstrates how social context impacts not only on how we come to think about sleep and understand it, but also how we manage or self-regulate our sleeping patterns.

## Introduction

Sleep, in one form or another, is a biological necessity for all living creatures. While the exact functions of sleep are still being determined and debated in the scientific community, the effects of going without sleep for extended periods of time have been fairly well characterised. Getting enough sleep is regarded as essential for our health and wellbeing (Ellenbogen 2005) and short sleep on a regular basis has been associated with the development a range of serious health problems, from obesity to cancer (Cappuccio *et al*. 2008, Thompson *et al*. 2011). Sleep deprivation has been associated with impaired cognitive performance, decreased productivity in the workplace and an increase in accidents and errors (Alhola and Polo-Kantola 2007, Barger *et al*. 2005, Gaba and Howard 2002), highlighting the social costs and consequences of poor sleep.

Concerns have been raised that, as we move further towards a global 24/7 society, the length of time we spend sleeping is being curtailed in favour of waking activities and social opportunities (Williams 2011). Studies have found that although people do value sleep and link it to their general wellbeing, at the same time, sleep is often not given priority, instead being viewed as a ‘disposable resource’ or ‘expendable luxury’ (Dzaja *et al*. 2005: 69–70). Sleep deprivation is thought to be commonplace in contemporary western societies and it is frequently claimed that on average, sleep duration is declining. Concerns about widespread sleep deprivation and the dangers of not getting enough sleep expressed by specialists in sleep science and sleep medicine have been picked up on and amplified through the media in recent years, transforming sleep from a personal matter and politicising it into a wider social problem of public concern (Boden *et al*. 2008, Kroll-Smith and Gunter 2005, Williams 2011).

Although a still fairly nascent field of study, research into the sociology of sleep has begun to shed light on the social, cultural and political dimensions of sleep. How, when, where and with whom we sleep is influenced by numerous social factors as well as by transitions across the life course (Williams *et al*. 2010). Recent studies have shown how the meanings and values people attribute to sleep and the importance they give to it is likely to be gendered (Hislop and Arber 2003, Meadows *et al*. 2008, Venn *et al*. 2008) and differ according to other socio-demographic and sociological factors including age (Venn and Arber 2011), socioeconomic status (Arber and Meadows 2011), occupation (Bryan 2011) and substance use (Nettleton *et al*. 2011). As such, socially appropriate ways of ‘doing sleep’ (Williams 2007) and temporal and spatial patterning of sleep may differ between social groups.

Recent large-scale surveys (Arber and Meadows 2011, Byran 2011, Chatzitheochari and Arber 2009) have drawn attention to the complex ways in which one's lifestyle, particularly in relation to work, impacts upon sleep. Shift work and long work hours (more than 48 hours per week), in particular, have been associated with short sleep duration (that is, sleeping for 6 or fewer hours per diurnal day) and poor quality sleep (Bryan 2011). Work hours have also been linked to the development of sleep problems, the quality of sleep people get and the amount of time people spend asleep each day. For example, a recent study found that men actively negotiate the amount of time they are going to spend sleeping in relation to the amount of work they have to get done the next day (Meadows *et al*. 2008), thus demonstrating the extent to which employment is a key factor in the social patterning of sleep (Williams 2011). Sleep loss and fatigue are therefore prevalent problems in the modern workforce, particularly as long work hours and shift work become more common (Chatzitheochari and Arber 2009, Stoller, *et al*. 2005). Consequently, the impacts that working patterns and workload have on sleep duration and sleep quality are of increasing salience as we move further towards 24-hour living in the UK.

Getting adequate sleep is considered to be important not only for physical health and psychological wellbeing but also for cognitive performance (Buboltz *et al*. 2009, Thacher 2008). Sleep quality and quantity are closely related to learning capacity and academic achievement, with sleep loss associated with a decline in neurocognitive and academic performance (Curcio *et al*. 2006). Large-scale surveys focusing on sleep in students have found that sleep problems, difficulties waking up and falling to sleep at ‘normal’ times and feeling tired are common in this social group (Buboltz *et al*. 2009, Thacher 2008). For instance, in a recent survey of sleep in over a thousand students attending a US university, over 60 per cent were categorised as poor sleepers. Students reported frequently taking prescription, over the counter and recreational psychoactive drugs to alter sleep/wakefulness. Poor quality sleep was linked to physical and mental health problems and students stated that emotional and academic stress negatively impacted on their sleep (Lund *et al*. 2009). In addition to engaging in academic work, socialising activities late at night and use of visual media (such as watching TV and surfing the Internet) have been related to delayed bedtimes (Asaoka *et al*. 2010). As economic pressures have increased, it is also common for students to work in paid roles, further cutting into their sleep time. All of these factors can impact on sleep quality and duration and lead to students sacrificing their sleep in order to prioritise the escalating around-the-clock demands of the 24/7 society.

Sleep, it seems, is something that is being cut back on, sacrificed or dismissed in different sections of society in order to prioritise the demands of the waking world. Sociological research into experiences of and expectations about sleep alerts us to how the broader social context, including lifestyle, work commitments, family responsibilities and the emotional labour (see Venn *et al*. 2008) attached to these social roles may influence sleep. Although there is much research to show that one's lifestyle and working patterns can have a significant influence on sleep duration and sleep quality, very few qualitative empirical studies have been conducted that focus specifically on the relationship between social context and subjective and embodied experiences of sleep. Building on previous research, the aim of this study was to conduct an in-depth and qualitative analysis of the sleeping practices and subjective experiences of sleep in two social groups, shift workers and university students.

Data are analysed in order to assess shift workers’ and students’ attitudes towards sleep and the impact of working patterns and demands of social life on sleep and wakefulness, and to explore how sleep(iness) and wakefulness are being managed in daily life in each of these social contexts. The findings contribute to the emerging body of literature on the sociology of sleep.

## Methods

Data are drawn from 25 semi-structured interviews that were conducted in 2008 as a part of a larger multi-method study^1^(Coveney 2010) investigating the medical and non-medical use of cognition-enhancing drugs in the UK. An aim of this study was to assess how pharmaceuticals that can extend wakefulness and allow people to cut back on sleep are perceived across different social domains. Three of the themes emerging from the interview data are of relevance to this article: attitudes towards sleep, impacts of working patterns and demands of social life on sleep and managing sleep(iness) and wakefulness in daily life.

In all 11 shift workers participated in the study. The participants were self-selected and recruited via a virtual space set up online via a social networking website with the group name ‘UK Shift Workers’. This was used to invite people who were resident in the UK and currently working shifts to take part in a short interview. As a common or organised space where people who work shifts (regardless of their profession or occupational role) gather to form a collective identity as shift workers was not identified, the creation of this virtual space enabled the researcher to assemble and access a group of people who would have otherwise been difficult or impossible to reach (Flick 2009). An implication of this sampling strategy is that the interview sample cannot be considered typical or representative of all UK shift workers. The interview sample consisted of a machine operator in a factory, an airport worker, a mental health nurse, a retail manager, a call centre operative, a delivery driver, two hospital-based nurses, two hospital-based doctors and a police officer. The respondents were aged between 21 and 53, seven were men and four were women. The occupational roles and shift patterns of the interview sample were mixed, as was the geographical location of the participants. On aggregate, the working hours of the shift workers who were interviewed spanned the full 24 hours of the day, 7 days a week at all times of the year.

In addition, 14 students were interviewed. The participants were randomly selected^2^ from a larger group of 80 students from one UK university who responded to an initial e-mail advertising the research and asking for volunteers to participate in a study about sleep and health. They were aged between 18 and 24, 10 were women and four men, and all were studying for undergraduate degrees at the time of interview.

Semi-structured interviews were used as a research tool in order to collect in-depth qualitative data on the meanings people attach to their experiences of sleep and wakefulness in each social domain, and explore the ways in which social structures and processes may shape these meanings (Bryman 2001). The interviews were either conducted face-to-face (21) or over the telephone (four) according to the preference of the respondent. Each interview lasted around an hour and was digitally recorded and professionally transcribed. The interviews were informal and conversational and the interviewees were given the freedom to describe their experiences and opinions in their own terms using their own words.

Interpretation of the data took an in-depth and qualitative approach. The qualitative software programme NVivo 8 was used to facilitate the organisation of the interview transcripts and to support a thematic analysis of the data (Bryman 2001). Each section of the data was systematically sorted and coded. Topics were indexed, collated and cross-referenced in order to identify recurring themes and patterns of ideas (Dingwall and Murphy 1998), leading to the establishment of ordered relationships between codes and theoretical concepts (Coffey and Atkinson 1996). Emerging themes were named, data included in each of the themes were re-read and refined and the specific details of each theme were organised to ensure that the themes were internally coherent and distinct from one another (Morse 1994).

## Findings

Firstly, I briefly discuss respondent's attitudes towards sleep. The main section of the analysis then focuses on experiences of sleep(iness) in the workplace and the university. Finally, I turn to the rituals and routines, and the array of tools and techniques used by the respondents in attempts to control sleep and promote alertness in their daily lives.

### Attitudes towards sleep

Although in general, the respondents were uncertain as to exactly what sleep was for; typically, sleep was valued and considered to be important for health, general wellbeing, appearance and physical and cognitive functioning. Both students and shift workers positioned sleep as something that was good for and needed by the body, an essential part of everyday life, a vital and natural period of time for the body and brain to rest and relax, repair, rejuvenate and recharge. As can be seen in the quote below, which is similar to the sleep narratives given by the men interviewed for Meadows *et al*.'s (2008) study, sleep was commonly spoken about in functional terms:

[Sleep is] very important, because I need it to function the next day … I think it's the time when the body, like, repairs and recharges itself. If I've had really bad sleep I can just see it in my face. I look really washed out and my skin gets all rumpled and that's, like, my indicator, that I need more sleep. (Nicola, student)

Eight hours’ sleep per 24-hour period was commonly referred to in normative terms as the recommended, right or full amount of sleep one ought to get. However, typically, sleep need was individualised, being constructed as something that was specific to each person. Respondents reported that they thought they needed between 4 and 10 hours sleep per night depending upon on how active they had been that day and what they had to do the next day. Despite acknowledging the importance of sleep, like Toby, most respondents said that, for various reasons, they would usually sleep for a shorter period than they thought they should do:

You want to do an 8-hour sleep, don't you? If you can. Well, five is, like, my regular – it's not enough but, like, it's all you can do. (Toby, airport worker)

In their accounts the respondents stressed the importance they placed on getting enough sleep by drawing on their own experiential knowledge of sleep deprivation and the experiences of friends and relatives. They discussed how lack of sleep could lead to illness, an impaired immune system, result in a poor diet, impaired daytime functioning and cognitive performance and have a negative effect on their appearance. Typically, the psychological, affective and cognitive impacts of sleep deprivation were emphasised in respondents’ accounts. Respondents reported feelings of mental fuzziness, lapses in attention and concentration, memory problems, inability to focus, trouble with complex decision-making, feelings of irritability and frustration, low mood, anxiety and depression. As Hannah says in the quote below, some respondents saw the impact of not getting enough sleep as transforming their personality and went as far as to say that without enough sleep they are not their normal self:

If I don't get sleep, the adequate, I'd say I need about 9 hours sleep, I feel groggy, I feel irritated, I just won't feel my normal self, a bit agitated and snappy. (Hannah, mental health nurse)

Respondents discussed the physical and emotional effects of lack of sleep as impairing their physical and cognitive performance. However, as Paul explains, the impact was thought to stretch far beyond this. Not getting enough sleep was also thought to influence the individual's ability to socialise with others and have a negative effect on interpersonal relationships and family dynamics:

I was really starting to get like frustrated with it when I first went onto nights, being tired all the time, and just taking it out on people that were around me and stuff – it makes you into not a very nice person. (Paul, factory worker)

Experiences of sleep and sleepiness as described by each group will be discussed further in the next section.

## Experiences of sleep(iness)

First, I discuss the impacts of shift work on sleep before focusing on the student data and students lifestyle on sleep patterns.

### Shift workers and broken sleep

The effects of shift work on an individual's sleep were commonly explained using biological understandings of the body. Respondents described how working shifts caused their body's ‘clock’ to fall out of sync with their shift pattern, resulting in the body feeling awake at times when the respondent wanted to sleep and vice versa. This was especially the case in accounts given by rotating shift workers, who thought that the constant change in the timings of their shifts made it difficult for them to get into a ‘proper pattern’ of sleep and wakefulness, leading to them feeling ‘tired all the time’.

All the shift workers who were interviewed described times when they would feel excessively tired and struggle to stay awake at work, especially when working nights. However, the way in which this behaviour was problematised differed between respondents and was to some extent related to their occupational role. For example, the two medical doctors described how they were able to use a specific space in the hospital designated for their use to rest and sleep by taking a power nap. For other respondents, sleeping during working hours, even during break times, was understood as illicit behaviour that could be dangerous for them professionally by leading to them losing their jobs but could also pose a danger to others by putting their lives at risk through negligence or causing accidents. Like Paul, several respondents told scare stories of colleagues who had fallen asleep at work, been caught and had lost their jobs:

It is pretty dangerous and I am sure I could lose my job if I got caught … I know a couple at work that have fell asleep before … one was woke up by a team leader, so he was sacked. (Paul, factory worker)

Those working in occupational roles where they were not directly responsible for the safety of others did not problematise workplace sleepiness and associated performance deficits to the same extent. Like Edie, they described having witnessed colleagues falling asleep at work and thought that if they were to lose concentration or fall asleep for a short period of time it would not be ‘a big deal’ and may even be a source of amusement for their colleagues:

I get a bit sleepy on the later shifts, but because it is quieter you don't really have to stay as alert … It wouldn't be a big deal [if I fell asleep] I don't think, and some people have done it before. So you just fit in with the crowd to be honest. (Edie, telephone operative)

As Moe, a delivery driver, explains, shift workers have to fit their sleep times around their work schedule and other social demands. Respondents described sleeping for shorter durations than they would like, catching up on sleep by taking a nap – a ‘planned period of sleep’ (Venn and Arber 2011: 201) – or sleeping for longer periods on their days off:

I find with working the shifts I work, you've got to sleep in the afternoon because if you don't, then you will feel knackered the next day, because you'd be going to bed at night, but still not getting enough sleep. [If] after work, you've had a couple of hours sleep then, I don't know, you can have a meal and go to bed later on. You need that bit in the afternoon. (Moe, delivery driver)

It was regularly stated that shift workers do not sleep well and this seemed to be taken for granted to a large extent. While some of those interviewed held 8 hours sleep as a benchmark, for the majority, 5 to 6 hours was generally perceived to be a more realistic duration and ‘enough sleep’ for them as individuals to be able to function adequately the next day. Conceptualising normal sleep as a ‘solid’ period of time taken during the night, shift work was thought to result in ‘broken’ sleep, which often had to be taken during the day. As can be seen in Karoline's account below, respondents considered that broken sleep had a more negative impact on the way they felt rather than changes to sleep duration or timing:

I would rather have 4–5 hours good sleep than I would 8 hours broken, because I find that if it is broken sleep it doesn't matter how long I have, I still feel as tired. So I always count myself as a good night's sleep is 4–5 solid hours. (Karoline, nurse)

In addition to trying to get one's body to sleep during the day when working a night shift, adjusting back to a ‘normal nocturnal pattern’ of sleep was something that had to be worked at:

I will make sure that I don't go to bed during the day if I know I've got to try and get myself back to a normal nocturnal pattern. I will try and make myself as tired as possible and then wait to go to bed until I'm absolutely shattered so I'm tired and therefore sleep all night long. (David, medical doctor)

Respondents discussed how fluctuating working hours often left them feeling exhausted, making it difficult for them to adjust back to normal daily rhythms outside the workplace in order to fulfil their other roles and responsibilities as parent, spouse and friend:

I do [see benefits of working shifts], but I think they're far outweighed by the costs for me … I've just finished working 12 days in a row and it just gets on top of you and, sort of, makes it really hard to just enjoy like, you know [your time off] … So, yeah, effect on family and social life. (Hamish, medical doctor)

To summarise, respondents described sleeping during the daytime, afternoons and evenings as well as during the night – sometimes work permitting and sometimes not. Their accounts reveal how sleep timing and duration are negotiated to fit around the demands of work time, as collectively they describe what Williams (2007) has referred to as ‘anarchic’ or ‘anomic’ sleep patterns, that is, sleep patterns that fall outside our normative ideals. Their accounts also signify the difficultly shift workers face in getting their bodies to perform in line with their social requirements, highlighting the disjuncture between the biological sleep drive and social sleep time (Wittman *et al*. 2006) that results from performing shift work. The limits of bodily agency are clear here as typically, these respondents considered themselves to have very little control over how well they sleep and how long they are able to sleep for.

### Students and flexible sleep

In the student data, the day and night worlds also seemed to merge, with respondents reporting both study time and social activities that spanned the full 24 hours of the day 7 days a week. Like Susie, it was usual for students to report working outside the traditional working day, studying late into the night or early in the morning, fitting their work around these other demands of the student lifestyle:

A few nights a week, I will be working until just before I go to bed because all my housemates are in bed, there's no distractions, no-one's calling you and there's not really much else to do … I don't really do all-nighters as such, but between 2 and 3am usually. (Susie, student)

A small number of students reported doing ‘all-nighters’; however, this pattern of working was commonly adopted by students when they felt under pressure, such as before a deadline or an exam. Alongside studying, having a good social life that included being an active member of clubs and societies, nights out and spending time with friends was also considered important, particularly for their emotional wellbeing and successful adjustment to living away from the family home. In contrast to the shift workers’ accounts and the lack of bodily agency they conveyed, the students gave a general sense of active agency in their perceptions that they were in very much in control of their sleep: ‘I can just stay up till whenever, so I can do work and then switch off when I feel like it (Daniel, student).

In addition to talking about sleep in functional terms, as discussed previously, there was also a strong sense in the student data that spending time sleeping was not a priority for them. Some students went as far as to refer to sleep as being a waste of their time, and it was commonly presented as something that, if cut back on, would give them more time for other activities. Despite acknowledging that they might need more sleep, like Becky, most said they usually got between 4 and 7 hours sleep a night, catching up by sleeping longer in their free time, on the weekends or in the afternoons when they did not have any other work or social commitments to attend to:

It's not often I'll deliberately go to bed when there's something else I want to do, because I want more sleep. I would usually just go out anyway and get through it. I'd just make sure I went to sleep early the next night or maybe have a nap in the afternoon or something. (Becky, student)

In a similar way to which the shift workers described having to fit their sleep time around their working patterns, students described modifying or customising their sleep patterns to fit around their studies and social engagements. Sleep was taken primarily during the night, but also during the mornings, afternoons and evenings in the form of short naps. Napping was discussed as an established practice and one that was engaged in without too much deliberation. Many respondents described napping during the day as a strategy for promoting alertness or catching up on sleep if they felt that they had not had enough sleep the night before. In this way, periods of sleep and wakefulness were typically thought of as flexible and under their control. They choose when they go to sleep and how long they sleep for, prioritising their waking life and negotiating their sleep time around both work-related and social activities. Accordingly, students’ sleep patterns could also be described as anarchic or anomic (Williams 2007), falling outside wider social norms and standards where normal sleep takes place in private, in a bed during the night and in one 8-hour block.

Despite conceptualising sleep timing and duration as being flexible and under their control, all the students interviewed reported having experienced difficulties getting to sleep and staying awake and alert at socially desirable times. Typically, these difficulties were not considered as being problems with their sleep *per se*, but as symptoms of other problems or factors such as stress, being in emotional turmoil or being too active before bedtime. Like Stephen, respondents often blamed themselves for the difficulties they had experienced, considering their behaviour to be a causal factor in the development of the problem:

I think it would be my own fault if I wasn't alert, it would be because I'd not been getting enough sleep and that's my decision. (Stephen, student)

While some students linked daytime sleepiness to a lack of sleep the night before, others felt that how much or how well they had slept the night before had little impact on how awake and alert they felt the following day. Instead, falling asleep or feeling sleepy in the day was constructed as a normal response to boredom, lack of activity or stimulation and socio-environmental factors:

I once got here and I was really, really tired and I thought I wouldn't survive the day. But then the lectures were really interesting and I was quite alert in the afternoon. When I am bored I usually get tired – a lot of people would. (Kerry, student)

As discussed in the previous section, students thought of sleep as being important, in terms of biological need and for their physical and cognitive functioning. However, despite this, their accounts reveal how they cut back on their sleep time in favour of work demands and social activities. While both groups describe their attempts to fit in an adequate amount of sleep per 24-hour period, the student's accounts reveal how this group have a greater opportunity for flexibility in their approach. This flexibility is the key in allowing them to adapt their sleeping patterns in ways that are favourable to maintaining a work–social life balance. In contrast to the shift worker data, biological control over the sleep–wake cycle and reference to the body's internal clock was not a significant feature of student discourse. However, limits to agency, in relation to sleep quality, sleep time and sleep duration were also evident in discussions of the accounts they gave. It is clear that we cannot easily make our bodies sleep or stay awake at socially desirable times, so therefore turn to a range of different strategies to aid us. These are discussed further in the next section.

### Managing sleep and wakefulness

Typically, students conveyed a sense of active agency in managing their states of sleep(iness) and alertness. Consequently, sleep problems were rationalised as a normal part of everyday life and were not pathologised or medicalised in their accounts. Similarly, despite the huge impact on sleep each of the shift workers described in their accounts, in general, those interviewed did not consider the problems they had experienced or were experiencing with sleep to be ‘real problems’ that would warrant medical attention. This can be seen in Hamish's account below:

I probably wouldn't seek medical help, because I would just say to myself, ‘Well, it's only going to be for this couple of weeks and then I've got a few days off, or then I've got a weekend’, so it would probably take quite a lot for me to ask for help … basically [I just manage it by myself]. (Hamish, doctor)

In a similar stance to that taken by Hamish, respondents from both groups said that they would seek medical advice for sleep problems only if the problem persisted for a prolonged period of time and they could not resolve it in other ways. As seen in the data extract below, many respondents were opposed to taking sleeping pills and thought this was all medical professionals would be able to offer them:

I wouldn't think of going to the doctor to be honest. They'd probably just say pretty much what's on my mind like ‘You shouldn't be doing this before’ and everything. I wouldn't want to end up on sleeping tablets, because I think it'd disrupt it even more, because if I then decide to go off them and whatever, I just don't want anything like that. It's not something I'd want. (Daniel, student)

Instead, most respondents said they would attempt to manage or cure their sleep problems themselves before going to see their doctor. They would do this in various ways: by looking on the Internet for advice, trying over-the-counter (OTC) remedies, speaking to a family member or trying to change social or environmental factors that they thought were causing the problem.

### Managing sleep

As with the women interviewed by Hislop and Arber (2003), a range of personalised strategies to aid the onset of sleep and promote alertness were discussed. Respondents reported reading, having a warm drink or a bath, doing some mild exercise or watching television as an aid to ‘switching off’, with varying levels of success. Some respondents reported self-medicating, using OTC pharmaceutical products, homeopathic remedies, antihistamines and alcohol as sedatives to help them to sleep when they were finding it difficult. Use of these substances was a prominent feature of the shift worker data, and also present (to a lesser extent) in the student data, although the rationale for use of these products did differ somewhat between groups. In the extract below a nurse who works a rotating shift pattern explains how she uses an OCT sleep aid to help her sleep after a night shift, justifying this action through appeal to the ‘design’ of her ‘body clock’:

I find that my routine is I have to go straight to bed as soon as I get in off my night shift and sometimes I might take things like Nytol to help me get to sleep because I find it so much harder to sleep in the day because my body clock isn't designed to sleep in the day. (Karoline, nurse)

In contrast, Joseph explains his use of an OTC product as being a way to help him to relax and calm down when he is feeling under stress in order to aid the onset of sleep:

I'm actually taking some Kalms pills after meals at the moment and that's basically to relax you and I have to admit that it's helping me a bit with my sleep at the moment, so it's basically putting my body in a calmer state, therefore my mind in a calmer state, so I have been able to get a bit more sleep since taking this medicine. (Joseph, student)

### Promoting wakefulness

All respondents thought that being busy, interacting and talking with people and keeping the body and brain active, although tiring, was the most important thing to keep them awake, alert and attentive in the workplace or while at university. Other ways of promoting wakefulness included taking breaks, getting a change of scenery, getting some fresh air, having a shower, browsing the Internet and watching television. Respondents reported drinking caffeinated drinks and energy drinks, eating sugary foods, taking caffeine pills and smoking cigarettes specifically to promote alert wakefulness:

I eat lots of chocolate to stay awake and drink lots of coffee and sometimes I take bottles of Red Bull and Lucozade and just hope that the patients just keep pressing their buzzers to keep us on the ball. (Kim, nurse)

Consumption of caffeinated products (drinks and caffeine pills) as an aid to promote wakefulness or to delay sleep was a prominent feature of the data. For example, in the extract below, Mike describes how he uses coffee as a wake-promoting substance, despite not liking the taste of it. He uses caffeine to extend wakefulness/delay sleep onset when he feels under pressure, forsaking sleep in order to get his work done:

I drink lots of caffeine … a few extra cups of tea or if I am feeling really pressured coffee – even though I can't stand the taste, but it keeps me awake more. That's usually what I do and just try and stay up longer, even if it is a case of getting not enough sleep – just to get [my work] done. (Mike, student)

One of respondents interviewed gave an account of a colleague who used illegal substances to stay awake during the night shift, but stressed this was not the norm in his place of work:

Everyone's tired but really they just keep going … Some have probably taken stuff … it's probably a rarity rather than a common thing … one lad was sacked last week … found him in the toilets, high as a kite … he was taking stimulants, he couldn't control them, he disappeared for hours hiding somewhere and eventually they checked the toilets, he was in one of the cubicles out of his head, so he got sacked on the spot. (Toby, airport worker)

Collectively, respondents reported using a wide range of strategies, techniques, technologies and practices to encourage, overcome or delay sleep(iness) and boost, promote or enhance wakefulness/alertness in their daily lives. Despite the different attitudes towards and experiences of sleep evident between both groups, the ways in which respondents described managing these cognitive states were strikingly similar.

## Discussion and conclusions

Recent studies have demonstrated how various social factors including age, lifestyle choices, working patterns, family structures and gender impact upon sleep patterns and practices (Hislop and Arber 2003, Meadows *et al*. 2008, Venn *et al*. 2008, Nettleton *et al*. 2011). Sociological studies have been informative about people's own definitions and normative expectations of what they consider to be ideal, adequate or enough sleep. However, sociological work on the experiences of sleep in different sections of society is still in the early stages. This article contributes to this emerging literature by exploring the sleeping practices and subjective experiences of sleep of two social groups: shift workers and students. It highlights the complex ways in which working patterns and social activities impact upon experiences and expectations of sleep in our wired awake world.

Shift work is one obvious area of modern life where biological and social dyssynchrony (Wittman *et al*. 2006) or the misalignment between biological and social times can be observed as working hours span the day–night divide. Although the label of shift worker does not signify a homogenous group and the working patterns and sleeping practices of those interviewed were varied, we can see in the shift workers’ accounts that they all have to work to fit their sleep time around their work time in attempts to foster an effective and workable sleep routine. Often they have little success in doing so as their bodies remain ‘recalcitrant’ (Nettleton *et al*. 2011), making it difficult for them to sleep in the daytime and leaving them feeling as though they are not able to spend enough time asleep. Shift workers (especially doing rotating shifts) cannot follow a set pattern of sleep and wakefulness, which results in ‘broken sleep’. Although the body might be tired and the individual might feel sleepy in the workplace, often the shift workers must go against their biological clock to stay awake and alert to do their job or face being sacked.

In Nettleton *et al*.'s (2011) study of the sleeping practices of heroin users, an inversion of sleeping patterns, so sleeping during the daytime rather than at night, was compatible with drug users living a life that is separate – both spatially and temporally – from non-users. This sleeping pattern had its benefits, allowing the drug user to ‘reside in their own space’ (Nettleton *et al*. 2011: 1370) and therefore helping them avoid the need to maintain troubled social relationships. In contrast, in shift workers accounts we can see that the effect of shift work on sleeping patterns has the opposite impact on the participants’ lives. The anomic sleeping patterns (Williams 2007) they are forced to adopt due to their work patterns are constraining at both a biological and social level. Lack of sleep impacts on their ability to function and perform in their role at work whereas socially, their sleeping patterns can disrupt family life by making it difficult for them to invest the time they would like into their social relationships and putting a strain on them. Fluctuating working hours also make it difficult for those who work shifts to adjust back to ‘normal’ daily rhythms outside the workplace in order to fulfil their other roles and responsibilities as parent, spouse and friend.

Students do not have the same constraints imposed on their time as shift workers. However, they too describe anomic sleep patterns and 24-hour lifestyles that prioritise social demands and activities over spending time sleeping. Students generally perceived themselves to be in control of their sleep and describe ‘customising’ (Williams *et al*. 2013) their sleep patterns by napping during the daytime and altering their sleep timing and duration at night to fit around the demands of their study and social lives. We can speculate that perhaps students normalise their sleep patterns once they leave the university context, entering paid work and experiencing further transitions across the life course, such as cohabitation, marriage and parenthood (Williams *et al*. 2010). However, the student data raise questions about the extent to which having an 8-hour block of nocturnal sleep is thought of as the only normal way of sleeping. As the data show, broken sleep and napping across the 24-hour period are becoming more normalised practices in different sections of society, perhaps signalling a wider shift or greater flexibility in perceptions and expectations about what normal sleep is in an increasingly 24/7 world.

In particular, the data around napping emphasise the complex politics of sleep (Williams 2011). Choosing to take a nap in your own time was generally perceived as an acceptable and everyday practice in the student data. However, perceptions of sleeping in the workplace were varied, ranging from it being an officially sanctioned practice in some occupations to a risky and illicit behaviour in others.

Although periods of sleep and wakefulness were understood as embodied experiences partly under biological control, the acts of ‘going to sleep’ and ‘staying awake’ described were acutely social. Collectively, the respondents’ accounts highlight what Williams and Crossley (2008) refer to as an ‘involuntary aspect of sleep’ and demonstrate the limits of agency here as the body often remains recalcitrant, defying the individual's efforts to enter or leave sleep at socially desirable times. Respondents described their daily rituals and routines, highlighting the ‘personalised strategies’ (Hislop and Arber 2003) they have developed in order to achieve sleep, and to wake up and maintain alertness at the times their work or social schedules dictate. Similar to respondents in other studies (Nettleton *et al*. 2011, Stoller *et al*. 2005), these respondents described how they would use and rely on various techniques and technologies to facilitate the transition to their desired state at the desired time. The analysis presented demonstrates how getting to sleep during the daytime and staying awake at night or for extended periods (for example, during long shifts) takes considerable negotiation, management, effort and planning on the part of the individual. It was evident across the data how much effort goes into getting some sleep or conversely, staying awake and alert in the workplace or at university at times that go against the dictates of our biology. The accounts given show the negotiations we all have to make between pragmatic concerns over getting enough sleep to be able to function sufficiently the next day and what is practical and possible in terms of the demands of our waking lives, working our requirements as best we can around the biological rhythms of sleep and wakefulness that are programmed into each of us.

With the increasing number of professions required to work shifts it may not be possible for those who cannot tolerate shift work to simply opt out. Williams (2011) has argued, drawing on the work of Hochschild (1989) and Venn *et al*. (2008), that when household chores, childcare activities and emotional labour are taken into account, in one sense we can all be considered as shift workers juggling the demands of working and family life, often at the expense of sleep. When we consider the 24/7 lifestyles of students and the ways in which sleep is already being customised to fit around our increasingly busy lifestyles, we can see how sleep is being relegated in the order of priorities, despite warnings from sleep experts about the health implications of this.

The impacts that sleep deprivation resulting from working patterns have on family life and personal identity have not been fully explored in this study and warrant further sociological attention. By uncovering experiences of and attitudes towards sleep, in addition to the rituals and routines and the array of tools and techniques already used on daily basis to control sleep and promote alertness in everyday life, we can gain a better understanding of the complex politics of sleep in contemporary society and how perceptions, behaviour and attitudes towards sleep are changing in the wake of an increasing social shift towards 24-hour living.

## Notes

1 Empirical work involved documentary and media analysis (Coveney *et al*. 2009), using 40 qualitative interviews with leading scientists and clinicians involved in sleep research and management and two potential user groups, students and shift workers.

2 Every fourth student was selected for interview from the list of 80 students who initially responded to an e-mail to participate in the study. Out of the 20 students who were contacted, 14 agreed to participate in the interview
